# Formulating hikikomori: social withdrawal in the digital age

**DOI:** 10.1192/j.eurpsy.2026.10327

**Published:** 2026-07-31

**Authors:** S. Kukurt

**Affiliations:** Psychiatry, Bezmialem Vakif University, İstanbul, Türkiye

## Abstract

**Abstract:**

In a world where digital connection is constant, many young people are quietly withdrawing from real-life engagement. Hikikomori, a condition first described in Japan, refers to severe social withdrawal and prolonged home confinement. However, once considered culture-specific, similar patterns are now seen globally, including among university students facing intense academic and social demands. In a recent cross-sectional study conducted across Turkey, 183 medical students were assessed using the Adaptive Behaviors Scale for Hikikomori Self-Report (ABS-H-SR), the Symptom Checklist-90-Revised (SCL-90-R), the Beck Depression Inventory, and a detailed sociodemographic form. Results indicated reduced class attendance, increased digital dependency, social isolation, and impaired interpersonal functioning, patterns that became more pronounced in the aftermath of the COVID-19 pandemic. These behaviours often stem from cognitive and emotional factors such as fear of failure, perfectionism, and avoidance. Digital environments may offer temporary relief, but they can also reinforce withdrawal. Integrating cognitive-behavioural, psychodynamic, and neurocognitive perspectives, this presentation conceptualises Hikikomori as a transdiagnostic expression of social dysfunction shaped by cultural and technological forces. Culturally sensitive therapeutic approaches (including stepped-care models and digital-era adaptations) will be discussed as ways to support reconnection and recovery in vulnerable student populations.

**Disclosure of Interest:**

None Declared

